# A Case of Ewing Sarcoma of the Mandible on ^18^F-FDG PET/CT

**DOI:** 10.22038/aojnmb.2019.13876

**Published:** 2020

**Authors:** Yasukage Takami, Fumitoshi Aga, Katsuya Mitamura, Takashi Norikane, Hanae Okuda, Yuka Yamamoto, Minoru Miyake, Yoshihiro Nishiyama

**Affiliations:** 1 Department of Radiology, Faculty of Medicine, Kagawa University, Japan; 2 Department of Oral and Maxillofacial Surgery, Faculty of Medicine, Kagawa University, Japan

**Keywords:** ^18^F-FDG, PET/CT, Ewing sarcoma, Mandible

## Abstract

Ewing Sarcoma is the second most common type of bone cancer in children. The dominant features of this malignant bone tumor are the tendency for rapid growth and metastasis. In addition, Ewing sarcoma of the mandible is extremely rare and can be mistaken for odontogenic infection. We report a 14-year-old girl who had had swelling, pain, and hypoesthesia in the left cheek for three weeks. She was diagnosed with pericoronitis initially, and then referred toour hospital due to worsening symptoms. CT and MRI revealed an expanding and destructive mass mainly in the left mandible. ^18^F-fluorodeoxyglucose (^18^F-FDG) positron emission tomography (PET) and fused PET/CT demonstratedincreased uptake in the mandibular lesion. Whole-body ^18^F-FDG PET images showed no abnormal activity except for the mandibular lesion. Histologic examination confirmed Ewing sarcoma. Although this tumor has an aggressive clinical behavior and rapid growth, early diagnosis can reduce patient’s morbidity, mortality and thus it is important to distinguish it from periodontal inflammation.

## Introduction

 Ewing sarcoma is the second most common primary bone cancer in children (1). Although Ewing sarcoma can occur in any part of skeleton, the most frequent locations are the long bones (58%), pelvis (20%) and ribs (7%) (2).The occurrence of Ewing sarcoma in the mandible is extremely rare (0.7% of all sites) (2). In the mandibular lesion, clinical symptoms such as swelling, pain and sensory disturbances are unspecific and can sometimes lead to misdiagnosis (3-6). 

 In addition, rapid growth and a tendency for metastasis are the dominant features of Ewing sarcoma; thus, mandibular involvement may be due to metastasis from another skeletal site (7). Several studies have demonstrated the usefulness of ^18^F-fluorodeoxyglucose (^18^F-FDG) PET/CT for staging, restaging and recurrence monitoring of Ewing sarcoma (8-9). Most case reports on Ewing sarcoma, involving the mandible have focused on the plain radiologic features, CT, and MRI. There are few descriptions of the findings of ^18^F-FDG PET/CT.

 Here, we reported a case of a patient with Ewing sarcoma undergoing ^18^F-FDG PET/CT, with increased uptake in the mandibular mass. 

## Case report

**Figure 1:**
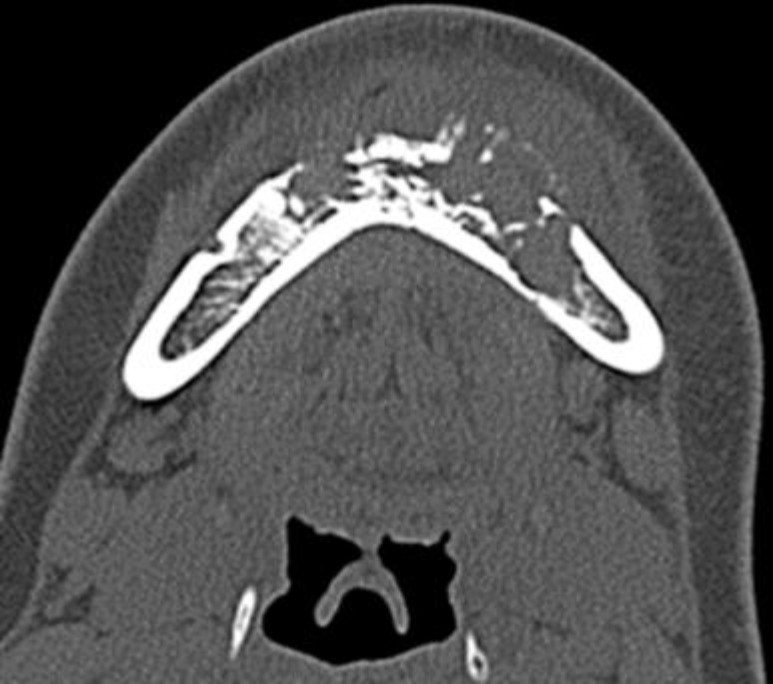
The bone window CT image shows a destruction of mandible

 A 14-year-old girl who had swelling, pain, and hypoesthesia in the left cheek for three weeks. She was diagnosed with pericoronitis initially, and then referred to our hospital due to worsening symptoms. Blood examination showed slightly elevated CRP (0.22 mg/dl). CT image showed an expansile lytic lesion in the mandible (Figure 1). 

**Figure 2:**
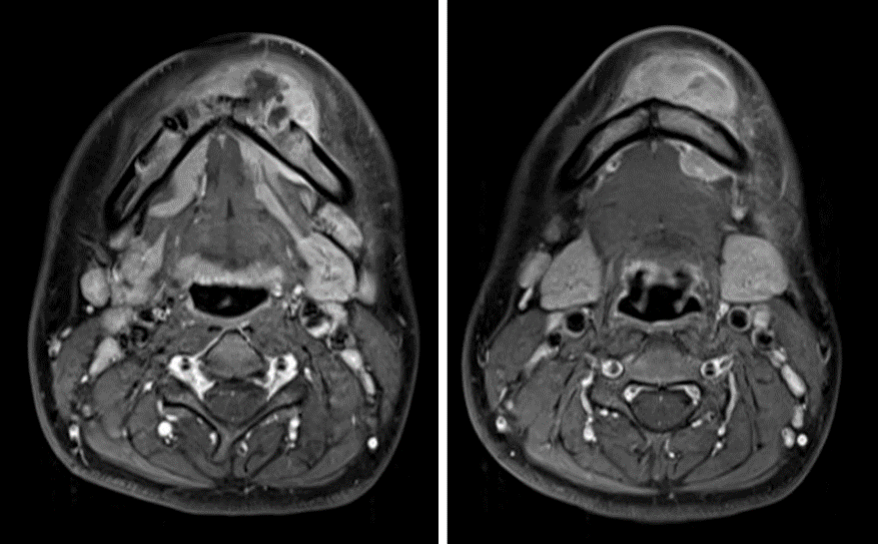
Transverse T1-weighted post contrast MR images reveal a destructive mandible lesion with extension to the adjacent soft tissue with inhomogeneous enhancement

**Figure 3:**
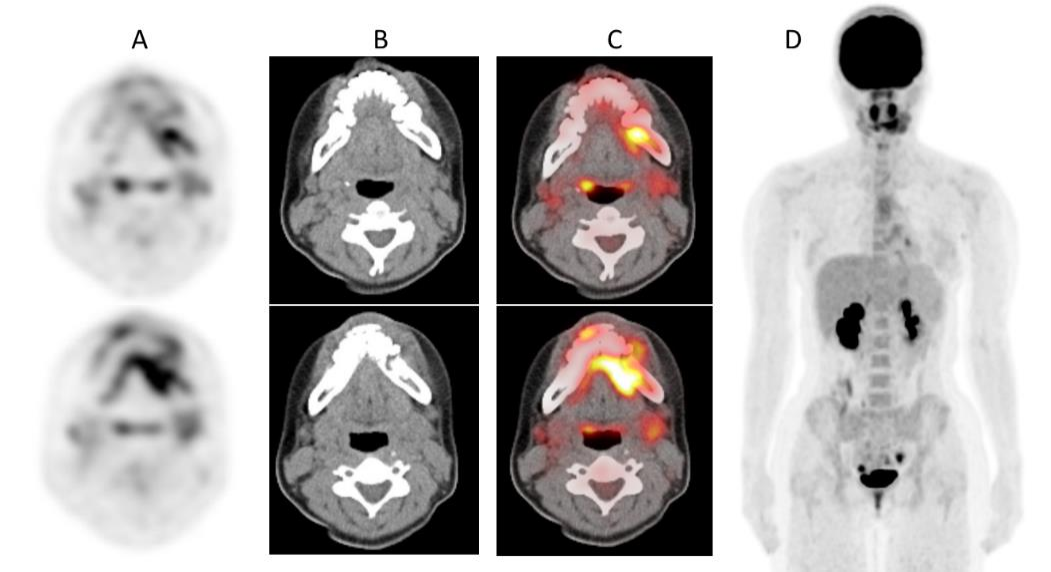
Transverse ^18^F-FDG PET (A), CT (B), and PET/CT fused (C) images show increased uptake in the tumor (SUV_max_=7.79). ^18^F-FDG PET maximum intensity projection image (D) reveals no other abnormal uptake suggestive of metastasis

 MRI images revealed destructive bone lesions with extension to the adjacent soft tissue mainly in the left mandible (Figure 2). ^18^F-FDG PET/CT was subsequently performed to evaluate distant metastasis. ^18^F-FDG PET and fused PET/CT images showed increased uptake in the mandibular mass (SUVmax=7.79) (Figure 3). Whole-body ^18^F-FDG PET images showed no abnormal activity except for the mandibular lesion (Figure 3).

**Figure 4:**
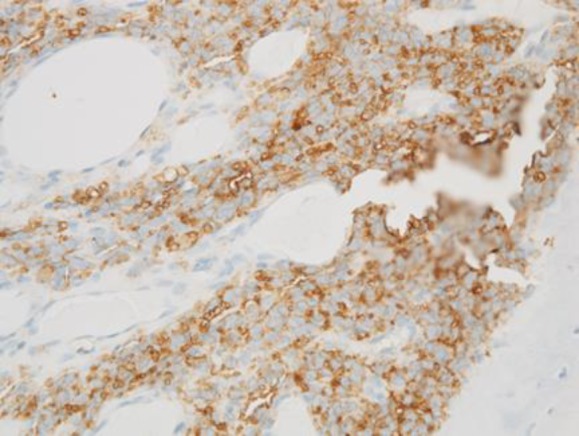
The tumor cells show strong membrane-staining pattern for CD99

 In this study, an incisional biopsy was performed. Histologic examination revealed a proliferation of small round to oval cells having hyperchromatic nuclei and scant cytoplasm, arranged in sheet-like patterns. Immunohistochemistry showed positive for CD99 (Figure 4), but negative for CD3, CD20, TdT and desmin. Reverse transcription-polymerase chain reaction analysis identified an Ewing sarcoma breakpoint region 1-ETS-related gene (EWSR1- ERG) fusion transcripts. The pathological findings indicated the diagnosis of Ewing sarcoma.

## Discussion

 Ewing sarcoma, which is very rare in the mandible, occurs most frequently in long bones (2) (2). Swelling, pain, and paresthesia may be first manifestations of Ewing sarcoma of the oral cavity, occurring also in dental infections (10). Therefore, Ewing sarcoma involving mandible case is often misdiagnosed as periodontal inflammation (6). In the present case, the patient was initially diagnosed with pericoronitis. 

 The general radiologic appearance of Ewing sarcoma is a mass accompanied by the destruction of bone (11). CT and MRI have been considered as the best imaging methods for evaluating the tumor (12). MRI is widely accepted as the imaging method for evaluation of the extent of the primary lesion. In this study, the mass was detected by MRI, and suggested the possibility of malignant pathology. However, it is difficult to distinguish Ewing sarcoma of the mandible from other mandibular tumors in children, such as osteosarcoma, rhabdomyosarcoma, malignant lymphoma, and metastatic carcinoma. 

 The present case showed the value of ^18^F-FDG PET/CT for staging of Ewing sarcoma in the mandible. Gupta et al. reported that the primary Ewing sarcoma family of tumors in patients with metastasis (mean SUV_max_=11.31) showed significantly a higher glucose metabolism than that in patients without metastasis (mean SUV_max_=6.84) (13). Conventional staging evaluation for Ewing sarcoma includes imaging of the primary tumor (typically with MRI), as well as imaging of the lungs with chest CT, bone scintigraphy to screen for bone metastasis, and bone marrow biopsies and aspirates (14). Recently, the use of ^18^F-FDG PET/CT to screen bone, soft tissue, and bone marrow metastases has been increasingly incorporated into the initial staging of the disease, as well as into surveillance both during and following completion of therapy (15).

 Ewing sarcoma is a radiosensitive tumor. Multimodality therapy consists of an initial biopsy, aggressive combination of surgery, chemotherapy and localized radiotherapy which are the therapeutic choices for Ewing sarcoma of the head and neck region and may result in a long-term survival. The prognosis of Ewing sarcoma is poor because hematogenous spread and lung metastases occur within a few months after diagnosis. The tumor burden is considered today as an important factor of prognosis (16). The conventional imaging like CT and MRI are highly recommended for an accurate evaluation of lesion’s prognosis on soft tissue and bone before initiation of treatment. ^18^F-FDG PET/CT was also used in this case to determine the extent and aggression of the tumor.

 In summary, a case of Ewing sarcoma in a young child highlighting diagnostic radiological feature in the mandible was reported. Familiarity with radiological findings will enable the clinician to rule out more common inflammatory lesions and narrow down differential diagnosis.
